# The impact of weather and increased atmospheric CO_2_ from 1892 to 2016 on simulated yields of UK wheat

**DOI:** 10.1098/rsif.2021.0250

**Published:** 2021-06-16

**Authors:** John W. G. Addy, Richard H. Ellis, Andy J. Macdonald, Mikhail A. Semenov, Andrew Mead

**Affiliations:** ^1^ Computational and Analytical Sciences, Rothamsted Research, Harpenden, Hertfordshire AL5 2JQ, UK; ^2^ Sustainable Agriculture Sciences, Rothamsted Research, Harpenden, Hertfordshire AL5 2JQ, UK; ^3^ Plant Science, Rothamsted Research, Harpenden, Hertfordshire AL5 2JQ, UK; ^4^ School of Agriculture, Policy and Development, University of Reading, Berkshire, UK

**Keywords:** atmospheric CO_2_, climate change, wheat grain yield, temperature, meteorological data

## Abstract

Climate change effects on UK winter wheat grain yield are complex: warmer temperature, negative; greater carbon dioxide (CO_2_) concentration, positive; but other environmental variables and their timing also affect yield. In the absence of long-term experiments where temperature and CO_2_ concentration were manipulated separately, we applied the crop simulation model Sirius with long-term daily meteorological data (1892–2016) for Rothamsted, Hertfordshire, UK (2007–2016 mean growing season temperature 1.03°C warmer than 1892–1991), and CO_2_ concentration over this period, to investigate the separate effects of historic CO_2_ and weather on simulated grain yield in three wheat cultivars of the modern era. We show a slight decline in simulated yield over the period 1892–2016 from the effect of weather (daily temperature, rainfall and sunshine hours) at fixed CO_2_ (294.50 ppm, 1892 reference value), but a maximum 9.4% increase when accounting for increasing atmospheric CO_2_ (from 294.50 to 404.21 ppm), differing slightly among cultivars. Notwithstanding considerable inter-annual variation, the slight yield decline at 294.50 ppm CO_2_ over this 125-year period from the historic weather simulations for Rothamsted agrees with the expected decline from temperature increase alone, but the positive yield trend with actual CO_2_ values does not match the recent stagnation in UK wheat yield.

## 1. Introduction

Temperature and carbon dioxide (CO_2_) atmospheric concentration have both increased on average annually from 1892 to 2016 [1]. These two factors show negative and positive effects, respectively, in experiments on wheat grain yield in the UK [2], and elsewhere [3]. In spite of continued variety improvement, wheat (*Triticum aestivum* L.) grain yields have stagnated since the mid-1990s in Europe [4] and the UK, with a mean value of 7.84 t ha^−1^ on UK farms for the period 1996–2017 [5], though with considerable inter-annual variation, following a substantial increase in annual yields over the previous 40 years [6]. This yield increase, from less than 3 t ha^−1^, was primarily a consequence of various agronomic improvements, including the introduction of short-strawed varieties, increased use of herbicides, fungicides and fertilizers [6], including applications of higher rates of N fertilizer. UK producers have striven to reduce inputs over the more recent period of yield stability, in order to both improve production efficiency and increase biodiversity [7]. However, wheat yield on the Broadbalk long-term experiment at Rothamsted, Hertfordshire, UK, has also remained relatively constant over this period on those plots receiving commercially relevant fertilizer applications [8] with no change in agronomy other than variety. These yield plateaus have occurred despite the continuing rise in atmospheric CO_2_ concentration [9] which experimentally increases photosynthesis [10], and so should increase crop growth and ultimately grain yield under UK field conditions [2].

Crops grown in elevated CO_2_ show a higher photosynthetic rate and also greater water-use efficiency [11]. Studies have indicated a 17% increase in yield with enrichment from 475 to 600 ppm CO_2_ [12], a 27% increase with enrichment from 541 to 620 ppm CO_2_ [9] and a 31% increase with doubling CO_2_ from 350 ppm to 700 ppm [13,14]. Overall, European agricultural systems are expected to show greater productivity under climate change combined with the continued development of crop technology and management [15], including wheat [16].

Global air temperature has been increasing more rapidly over the last 30 years than earlier in the last 150 years [1], the average in 2016 being 1.43°C above that for the twentieth century [1]. Similarly, the UK 2007–2016 decadal average was 0.8°C above the 1961 to 1990 average [17]. An increase in temperature tends to reduce crop yield because it shortens the duration of the crop growing season, a major determinant of yield [18]. In addition, high temperatures at flowering can reduce the potential number of wheat grains that contribute to crop yield [19,20] and warmer temperatures from then onwards depress mature seed dry weight [21].

Climate change will influence agriculture and global food security through altered agroecological environments [22]. The impact of change in one environmental variable, such as temperature, on crop production over time may be confounded by simultaneous changes in others. For example, in field experiments [2], grain yield in wheat was greater at elevated CO_2_ but reduced by warmer temperatures, with the increase from doubling CO_2_ negated by a 1.0–2.0°C increase in mean seasonal temperature. Other environmental variables have long been known to affect wheat yield, however, particularly rainfall [23,24]. Hence, the effects of change in CO_2_ on yield needs to be considered in the context of all changes in weather in the production environment.

**Figure 1.**
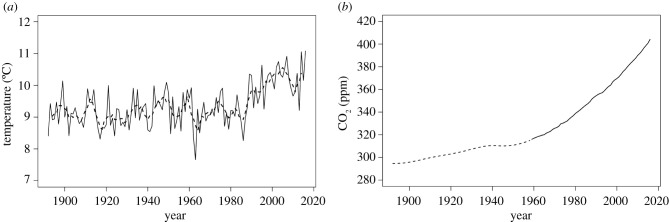
(*a*) The annual mean temperature, calculated from daily maximum and daily minimum temperatures, at Rothamsted, Hertfordshire, UK, from 1892 to 2016. Each year, *i*, represents data for a harvest year from October (year *i*-1) to September (year *i*). The solid black line represents the annual mean temperature and the dashed line the 5-year average. (*b*) The yearly atmospheric CO_2_ concentration was that estimated from ice core data between 1892 and 1958 [26] (dashed line) or that recorded at the Moana Loa Observatory between 1959 and 2016 [27] (solid line).

The Rothamsted Meteorological Station (RMS), a centennial observing station in Hertfordshire, UK [25], has recorded daily rainfall, temperature and sunlight (sunshine hours) together since 1892. Temperatures there increased markedly from around 1980 onwards (figure 1*a*), with 2007–2016 mean (October to September wheat growing season each year) 1.03°C above the 1892–1991 average. The estimated atmospheric CO_2_ in 1892 was 294.50 ppm [26]. In April 2014, it crossed the 400 ppm threshold [27] (figure 1*b*).

In the absence of long-term experiments allowing the separate manipulation of CO_2_ concentration and weather, and hence the partitioning of observed yield variability due to these different environmental inputs, we conducted a simulation study. This combined the Rothamsted historical weather data [28] with reconstructed [26] and measured [27] atmospheric CO_2_ data within Sirius [29], a process-based crop simulation model, to separately estimate the effects of weather and change in atmospheric CO_2_ from 1892 to 2016 on simulated wheat yield under uniform agronomy. Our objective was to determine the pattern of variation in simulated continuous wheat yields under constant agronomy at this one site (Rothamsted) during this 125-year period in order to identify the combined and separate contributions of historic variation and trends in weather, and of the rise in atmospheric CO_2_ concentration, to this pattern.

The Sirius model was chosen because it has been extensively tested and validated and has performed well under diverse climatic conditions across Europe, North and South America, Australia and New Zealand [16,30–35]. The modelled responses to increased temperature and CO_2_ concentration were validated previously against Free-Air CO_2_ Enrichment (FACE) experiments [36–38] and tested in several AgMIP (Agricultural Model Intercomparison and Improvement Project) studies [39–41] considering a greater range of weather environments than those observed at Rothamsted over the 125-year period of this study.

To assess the impact of changes in CO_2_ concentration, two sets of simulations were considered for each season, a reference simulation where CO_2_ was fixed at the 1892 level of 294.50 ppm [26], and a test simulation with the observed value of CO_2_ applied each year. Simulations were included for three short-strawed varieties of the modern era with contrasting physiological traits and different rates of phenological development (Avalon, Claire and Mercia; electronic supplementary material, table S1), for which Sirius had been previously calibrated [29,42–47], to acknowledge potential genotype-by-environment interactions influencing yield responses. The traits and characteristics differing between varieties include the length of the accumulated day-degree phyllochron period, the development response to day length, the thermal time from anthesis to start of grain fill, the vernalization rate, the maximum flag leaf area and the minimum and maximum numbers of leaves. All other parameters were common across these three varieties.

## 2. Results

**Figure 2.**
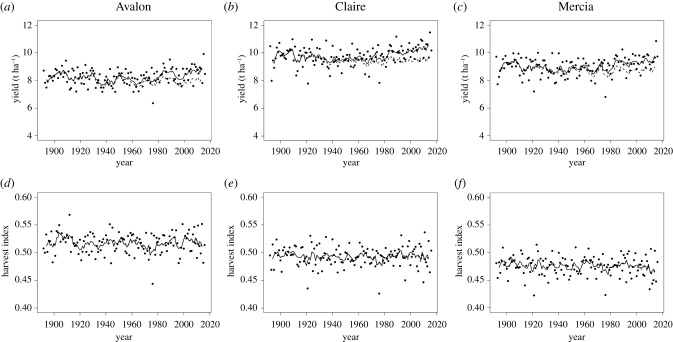
Simulated wheat grain yield ((*a*), (*b*) and (*c*)) and harvest index ((*d*), (*e*) and (*f*)) generated by Sirius for Rothamsted, Hertfordshire, UK from 1892 to 2016 for the varieties Avalon ((*a*), (*d*)), Claire ((*b*), (*e*)) and Mercia ((*c*), (*f*)) with weather from the RMS dataset and the observed increasing atmospheric CO_2_ concentration over this period (•). The solid lines provide 5-year rolling means of the simulated yields (top) and harvest index (bottom). The dashed lines (top) provide 5-year rolling means of simulated grain yields where CO_2_ was fixed at the 1892 reference value of 294.50 ppm.

Simulated wheat grain yields (for Rothamsted weather) with increasing CO_2_ concentration (figure 1*b*) from 1892 to 2016 showed considerable inter-annual variation in response to weather variability (figure 2*a–c*), and a slight, but statistically significant, average increase of 0.00336 t ha^−1^ per year (s.e. = 0.000894, d.f. = 371, *p* < 0.001) over the whole 125-year period, with no evidence of different average rates of annual increase between varieties. There were consistent differences between varieties on average, however, with Claire the highest-yielding variety, with a mean of 9.79 t ha^−1^ compared to means of 9.04 t ha^−1^ and 8.25 t ha^−1^ for Mercia and Avalon, respectively. By contrast, simulated wheat grain yields (for Rothamsted weather) with a constant CO_2_ concentration of 294.50 ppm showed a slight, but statistically significant, average decrease of 0.00265 t ha^−1^ per year (s.e. = 0.000842, d.f. = 371, *p* = 0.002) over the whole 125-year period, again with no evidence of differences in this decline between varieties. As for the increasing CO_2_ scenario, there were consistent differences between varieties on average, however, with Claire the highest-yielding variety with a mean of 9.51 t ha^−1^ compared to means of 8.78 t ha^−1^ and 8.02 t ha^−1^ for Mercia and Avalon, respectively. However, some care is needed in interpreting these trends with the year (where the term year is a proxy for the weather for each growing season), since the differences between years include the impact of the complex variability of weather factors, resulting in the considerable inter-annual variation in simulated yields which dominates these slight trends. Hence these simple regressions should only be considered as indicative of the trend observed across the simulations plotted against year, and of a possible association with, especially, average temperature, which also increases across the 125-year period. We therefore assess the impact of the increase in CO_2_ concentration by considering the (percentage) difference in simulated yields between the fixed and increasing CO_2_ scenarios, eliminating the impact of this inter-annual variation.

The difference in yields between simulations with increasing and fixed levels of CO_2_ became increasingly evident from the mid-twentieth century (figure 2*a*–*c*), with only very small differences in decadal means for the 1900s (9.32 t ha^−1^ for fixed CO_2_ concentration compared to 9.35 t ha^−1^ for varying CO_2_ concentration, averaged across varieties), and with differences steadily increasing by the 1940s (8.68 t ha^−1^ compared to 8.81 t ha^−1^), 1970s (8.62 t ha^−1^ compared with 8.91 t ha^−1^) and 2000s (8.74 t ha^−1^ compared with 9.38 t ha^−1^). For increasing CO_2_ levels from 1892 to 2016, Avalon had the largest mean simulated harvest index of 0.52 compared to 0.49 and 0.48 for Claire and Mercia, respectively (figure 2*d*–*f*). There was no trend over time for the simulated harvest index (in agreement with previous experimental observations [14]), nor any impact of the different patterns of CO_2_ (data not shown), suggesting the allocation of biomass to the grain and non-grain specific plant growth was the same over time and across CO_2_ scenarios.

**Figure 3.**
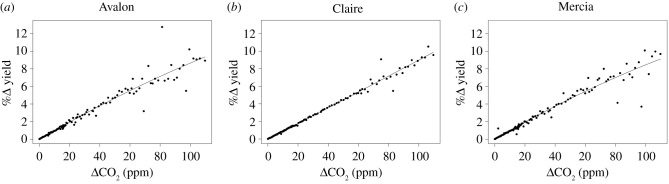
Percentage difference in wheat grain yield for Avalon (*a*), Claire (*b*) and Mercia (*c*) in response to increase in CO_2_ concentration (from 294.50 ppm) (•) derived from simulations by Sirius for Rothamsted, Hertfordshire, UK from 1892 to 2016 (further details as figure 2). The fitted weighted regression lines are constrained through the origin and are quantified in table 1.

The 1991–2016 25-year mean grain yield from simulations with increasing CO_2_ was 8.50, 10.18 and 9.38 t ha^−1^ for Avalon, Claire and Mercia, respectively, compared to 7.92, 9.48, 8.75 t ha^−1^ with the 1892 reference CO_2_ value (294.50 ppm), a mean difference of 0.64 t ha^−1^ (+7.3%). The percentage increase in simulated yield for actual CO_2_ each year over the 1892 reference value was modelled against the increase in CO_2_ as a quadratic relationship using a weighted regression due to the systematic increase in yield variability with greater CO_2_ (figure 3). The curvilinearity detected suggests that the benefits of increased CO_2_ were greater at lower rather than higher levels of atmospheric CO_2_ in Avalon and Mercia, but not in Claire where the relationship was almost linear with a very limited, but positive, curvature (table 1). Hence, Claire benefitted marginally more from the increase in atmospheric CO_2_ than Avalon or Mercia: increasing the atmospheric CO_2_ concentration from the 1892 baseline of 294.50 ppm to 404.21 ppm for 2016 resulted in 9.36, 9.87 and 9.12% greater simulated grain yields for Avalon, Claire and Mercia, respectively.

**Table 1.** The estimated main effect of increasing atmospheric CO_2_ (from 294.50 ppm to observed levels) on wheat grain yield in the varieties Avalon, Claire and Mercia at Rothamsted, Hertfordshire, UK from 1892 to 2016 provided by comparing the reference and test simulations by Sirius using weather from the RMS (figure 2). The fitted curvilinear relationships and the simulated observations for each year are shown in figure 3. $\mathrm{Weights}=\;1/r_{i}^{2}$.

coefficient	estimate	s.e.
CO_2_: Avalon	0.0998	0.0001
(CO_2_)^2^: Avalon	−0.0001328	0.0000019
CO_2_: Claire	0.0885	0.0001
(CO_2_)^2^: Claire	0.00001335	0.0000019
CO_2_: Mercia	0.0999	0.0001
(CO_2_)^2^: Mercia	−0.0001516	0.0000052

**Figure 4.**
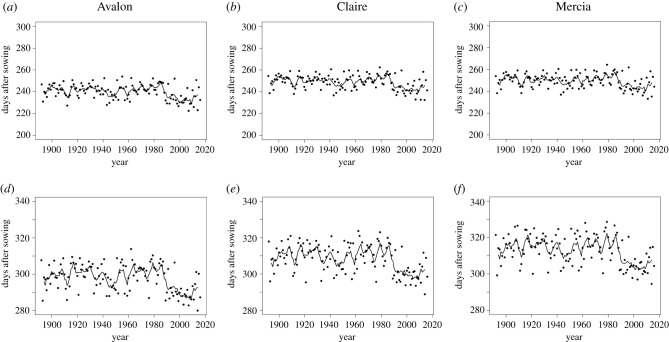
Durations (days) from sowing to anthesis ((*a*), (*b*) and (*c*)) or to harvest maturity ((*d*), (*e*) and (*f*)) simulated by Sirius in wheat for the varieties Avalon ((*a*), (*d*)), Claire ((*b*), (*e*)) and Mercia ((*c*), (*f*)) for Rothamsted, Hertfordshire, UK from 1892 to 2016 with weather from the RMS and the increasing atmospheric CO_2_ concentration over this period (•). The solid lines provide 5-year rolling means of these durations.

The increase in temperature at Rothamsted between 1892 and 2016 (figure 1*a*) reduced durations to the start of anthesis (figure 4*a*–*c*) and to maturity (figure 4*d*–*f*) in both the reference and test simulations. The patterns of results indicated a step change between 1980 and 2000. The 100-year mean (1892 to 1991) simulated anthesis dates for Avalon, Claire and Mercia were 242, 250 and 251 days after sowing (DAS), but 8 (234 DAS), 7 (243 DAS) and 7 days (244 DAS) earlier, respectively, for the subsequent 1992–2016 25-year mean. Durations to maturity were affected similarly: the 1892–1991 means were 300, 310 and 315 DAS, respectively, with the 1992–2016 means 10 days earlier for all varieties (290, 300 and 305 DAS, respectively).

## 3. Discussion

In contrast with investigations in modified UK field environments with irrigation, in which warmer temperatures reduced both crop duration and yield and greater CO_2_ increased yield [2], this simulation study for Rothamsted, UK, in which rainfall varied according to that recorded at the site for each year, indicated reduced crop duration (figure 4) with warmer temperature (figure 1*a*) but only a marginal reduction in yield alongside considerable inter-annual variation (figure 2). In particular, the marked reduction in simulated crop duration from around 1990 was not replicated for simulated yields, probably because of reduced water stress. A result of the warmer temperatures shortening the growing season is that cumulative transpiration up to anthesis is reduced, leaving more water in the soil available for wheat growth. In turn, this reduces the effect of water stress at anthesis and during grain filling on grain yield. Hence, in water-limited summer conditions in the UK, the better availability of soil water at anthesis and during grain filling may compensate for the direct impact of increased temperatures of the duration of the growing season. Simulated yield did increase with greater CO_2_ (figure 2), despite any counteracting effects of increased temperature.

This was a simulation study using a complex model but with most parameters fixed across the treatments considered. An assumption of uniform agronomy enabled fair comparisons between the treatments of interest but is not realistic: for example, the increase in yield with an increase in atmospheric CO_2_ (figure 2*a*–*c*) implies a need for more fertilizer in order to maintain grain protein in the crop [48] and soil fertility for the subsequent crop, by replacing the nutrients removed by the harvested crop [49,50]. If so, this would tally with the record rotational wheat yield on the Broadbalk long-term experiment at Rothamsted of 12.99 t ha^−1^ in 2014 for a new variety, Crusoe, in a first rotation with large inputs of N (288 kg N ha^−1^), compared to a mean of 6.34 t ha^−1^ for continuous wheat with 192 kg N ha^−1^ between 1996 to 2016 (electronic supplementary material, figure S1). Similarly, the greater yield potential, associated with the increase in atmospheric CO_2_ and changes in weather patterns may, in practise, affect crop vulnerability to pests, diseases and weeds [51], reducing yield below the yield potential simulated [52,53] unless compensated by additional crop protection measures. A previous study of the relationship between the historic UK mean annual wheat yields and CO2 concentration [54] identified how observed yield increases in the second half of the twentieth century exceeded what would have been expected given earlier trends with increasing CO_2_ concentrations, reflecting the importance of also considering agronomic improvements when modelling real-world responses. The uniform agronomy assumed for this study, with the model not accounting for any differential effects of climate change on weeds, pest and diseases, and hence on the interactions of these factors with crop growth and development, enabled the assessment of the impacts of changes in weather and CO_2_ concentration on yields in isolation from the effects of these agronomic factors, but also identifies the need for the model to be extended to better reflect the whole cropping system.

The mean increase in simulated grain yield from the increase in atmospheric CO_2_ concentration between 1892 and 2016 for the three varieties considered (Avalon, Claire, Mercia) was 9.42% using the Rothamsted long-term weather dataset (figure 3). Field validation of crop models under late nineteenth and early mid-twentieth-century CO_2_ concentrations is not possible. The conservative assumptions in Sirius with regard to the response to CO_2_ (from FACE experiments where ambient values were greater than 294.50 ppm CO_2_ [36–38]) at low concentrations imply that any putative error in the current study would be to underestimate the uplift in yield from the increase in CO_2_ concentration between 1892 and 2016. The curvilinear relationship between the yield benefit and the increase in CO_2_ for Avalon and Mercia was expected over the simulated range [55,56]. The more linear response for the variety Claire was unexpected. While the difference in curvilinearity was small, the potential implication that certain traits may support improved adaptation to greater atmospheric CO_2_ concentrations requires further consideration. The Sirius model parameters for Avalon, Claire and Mercia differ in a number of phenological and morphological characteristics (electronic supplementary material, table S1). In particular, Claire has the longest accumulated day-degree phyllochron period and also the lowest minimum and maximum possible leaf numbers specified for the Sirius model, with durations to anthesis similar for Claire and Mercia (figure 4). We speculate, therefore, that the greater leaf longevity of Claire may be pertinent to its superior simulated response to the highest atmospheric CO_2_ concentrations.

Agriculture, forestry and other land use are, simultaneously, both sources and sinks of greenhouse gases, accounting for 23% of total net anthropogenic greenhouse gas emissions [57]. Improved efficiency in food production, particularly in terms of yield per unit area, can not only benefit food security but it also has the potential to contribute to climate change mitigation and adaptation and, further, through reducing the demand for crop land, reduce desertification and other land degradation [57]. Hence, investigation (virtual and real) of those wheat varietal traits, such as leaf longevity, that may improve the crop's responsiveness of yield to higher ambient CO_2_ concentrations, merit further attention. Given the impact of greenhouse gas emissions, particularly nitrous oxide, with its high global warming potential, associated with the production and use of nitrogen fertilizers [57], the importance of greater leaf longevity would be amplified in scenarios where crop management practices limited nitrogen application rates. Wheat grain yield is highly responsive to nitrogen fertilizer in the UK and the curvilinear response, and the maximum asymptotic yield, show considerable sensitivity to the weather at several specific periods during the growing season [58].

The effect of increased CO_2_ from 1892 to 2016 on simulated wheat yields (figure 3) occurred during a period of warming at Rothamsted (figure 1*a*). A greater benefit of CO_2_ on yield over this period may have been observed without warming, since a temperature increase of 1°C may reduce yield by 3.5% [59]. The actual rise in temperature over this period at Rothamsted was about 1.2°C. Our simulations included the effects of daily rainfall and sunshine varying between years, not temperature alone, and detected high inter-annual variability (figure 2*a*–*c*) but only a marginal, though statistically significant, declining linear trend for yield over this period for scenarios at the 1892 CO_2_ concentration reference value. However, we caution against any strong inference being made about this decline with year, both because the year is just a proxy for the complex inter-annual variation in weather, and because using the same weather data for both the varying and fixed CO_2_ scenarios introduces a lack of independence between the two series of simulated outputs. Hence the focus in this study on analysing the percentage difference in simulated yields between the two scenarios, adjusting for the inter-annual variation in weather, and allowing a direct assessment of the impact of increasing CO_2_ concentrations. Additionally, warmer temperature shortens the growing season (figure 4) which in turn reduces the effect of water stress at anthesis and during grain filling on grain yield. Hence in water-limited conditions the impact of the shorter growing season may be compensated by the reduction in water stress.

Without the increase in atmospheric CO_2_ from 1892 to 2016, wheat yields would now be lower but the increase in simulated yield at Rothamsted (figure 2) over the last quarter century does not tally with the observed stagnation in UK wheat grain yield [5,8]. The latter may in part reflect a drive by growers to reduce inputs and increase biodiversity [7], and elevated ozone concentrations could also have contributed [60], but it is also important to remember that all models are simplifications of the real world. Simulation of observed yield variation at the farm and larger scales is difficult not least because factors such as weeds, pests and diseases are generally excluded from crop simulation models [37] together with the complex economic, political and social factors that influence farmers' decisions. Over the period of the current study, actual yields for continuous wheat with no inputs at Rothamsted have been largely stable with no long-term trend whereas mean UK farm yields are now around four times those of the late nineteenth century due to the widespread adoption of improved agronomy [6] (see figure 1.3 of the reference). As crop simulation models are used widely to estimate climate change impacts, an important area of model development is to extend these crop simulation models to incorporate the impacts of climate change on weed, pest and diseases, together with the interactions of these factors with crop growth and development under climate change, and to consider how the economic, political and social factors influencing farmers’ decisions can be accounted for. We therefore caution that estimates of benefits to wheat yield with a further rise in CO_2_ concentration, whether derived from real or virtual investigations, may not necessarily be realized by farmers in practice.

## 4. Methods

### 4.1. Data and model

The processed-based wheat model Sirius [29] was applied to observed weather (rainfall, temperature and sunlight) and estimated atmospheric CO_2_ data at Rothamsted from 1892 to 2016 to simulate continuous wheat yield data for three varieties from the modern era with contrasting physiological traits. A simulation of wheat yields from 1892 to 2016, where CO_2_ was increasing over time, was compared to a simulation where CO_2_ was fixed at a reference 1892 level (294.50 ppm), with the same weather data used for both sets of simulations. In Sirius, radiation use efficiency (RUE) is proportional to atmospheric CO_2_, with an increase of 30% for doubling in CO_2_ compared with the baseline of 350 ppm, which agrees with the meta-analysis of different field-scale experiments on the effects of CO_2_ on crops [9]. A similar response is used by other wheat simulation models, such as CERES [36] and EPIC [61].

The varieties chosen for this study were Avalon, Claire and Mercia, each calibrated for Sirius using experimental data [32,44,47,62]. Avalon, Claire and Mercia are modern winter wheat cultivars with contrasting physiological traits and different rates of phenological development (electronic supplementary material, table S1), though none of these varieties have ever been grown on the Broadbalk long-term experiment. Mercia has a higher potential leaf size compared to Avalon and Claire. Claire has the longest accumulated day-degree phyllochron period compared to Avalon which has the shortest. Claire has the lowest possible maximum leaf number of 18 compared to 24 for both Avalon and Mercia. Avalon has a larger day-length response compared to Claire and Mercia.

Sowing date was set to 15 October, soil type (identified for the Broadbalk long-term experiment [63] as ‘clay loam to silty clay loam over clay with flints' and specified in the Sirius soils database as ‘medium silty over clay drift with siliceous stones’) and data for the Rothamsted site, and the initial conditions, water deficit and soil inorganic N (30 kg N a^−1^) were re-set to original values each year. At this site, the initial soil moisture conditions do not differ markedly between years and field capacity will be reached during late autumn in each year, so that soil moisture will not have a marked impact on crop development in the first few months after sowing. The nitrogen application date was set to 15 April with a single application of 192 kg N ha^−1^ each year. Water and nitrogen limitations were activated in all Sirius [29] simulations.

Daily rainfall, maximum temperature, minimum temperature and hours of direct sunlight from the RMS for 1891 to 2016 were used [28]. (Note that there was a change in rain gauge in 2004 which recovered 10% more rainfall. Earlier records were adjusted by this value.) To accompany the 1892 to 2016 weather dataset, CO_2_ data from two sources were used: atmospheric reconstruction, derived from ice cores for the period 1892 to 1958 [26] and measured values from the Moana Loa Observatory for 1959 to 2016 [27]. Two simulations using the RMS dataset were conducted: one using the RMS data where CO_2_ was varied (figure 1*b*) and a second with the same RMS weather data but with CO_2_ fixed at the 1892 reference level (294.50 ppm). The percentage increase in grain yields from the simulations with varying CO_2_ levels relative to those with the reference CO_2_ level was calculated for each year and variety as

$$\text{\%}\Delta y_{i}=100\times \left(\frac{y{\left(CO_{2}\;Varying\right)}_{i}}{y{\left(CO_{2}\;Reference\right)}_{i}}-1\right),$$

where *y* is grain yield (t ha^−1^) and *i* is year (of harvest) from 1892 to 2016.

### 4.2. Statistical analysis

Quadratic regression analysis through the origin was used to investigate the curvilinear relationship between the percentage increase in yield between simulations with varying and fixed CO_2_ $\text{\%}\Delta y_{i})\;$, the corresponding increase in atmospheric CO_2_, for all years from 1892 to 2016. Weighted regression was used because of the systematic increase in variability as percentage difference in yield increased. The weights for each observation of the analysis were the reciprocal of the squared residuals $\left(1/(r^{2})\right)$ from a fitted unweighted quadratic regression through the origin. A ‘regression with groups' approach was applied, with variety included as a factor in the model to allow assessment of the need for separate parameter estimates for the three varieties. This enabled the assessment of whether there was a common relationship across varieties, or whether different relationships were appropriate for each variety.

Linear trendlines were fitted to the simulated yield data for both the varying and fixed CO_2_ scenarios against year (from 1892 to 2016), using a ‘regression with groups’ approach to allow assessment for differences in the trendlines between varieties. The purpose of this analysis was to identify if there were long-term increases or decreases in the simulated responses for the different scenarios and whether these varied between varieties. However, formal inference from these fitted lines should be avoided, primarily because the year is a proxy for the complex pattern of weather variation between years. Hence year cannot be considered a reliable predictor of any trends. In addition, as the same weather data are used in both scenarios, the residuals lack independence, and as the responses are, by design, very similar in the early years, any difference in the fitted slopes is heavily influenced by the responses in the more recent years. Direct comparisons between the results for the contrasting scenarios should therefore only be made via the weighted regression for the computed percentage yield differences against the observed CO_2_ concentrations described above.

Across all simulations between 1892 and 2016 for the three varieties, two variety-by-year combinations provided potentially anomalous results regarding the impact of the different levels of CO_2_. In 1956, the simulations for Avalon provided a marginally lower yield from the varying (i.e. greater concentration) CO_2_ scenario of 7.739 t ha^−1^ compared to 7.744 t ha^−1^ from the reference (fixed) CO_2_ scenario. This marginal decrease in grain yield from the greater CO_2_ concentration appeared to derive from more of the simulated growth of the crop going into non-grain biomass, with total biomass for the CO_2_ varying scenario of 15.52 t ha^−1^ compared to 15.36 t ha^−1^ from the reference CO_2_ scenario. In 1903, the simulations for Mercia provided a marginally lower total biomass with the varying CO_2_ scenario than from the reference CO_2_ scenario (18.81 t ha^−1^ compared to 18.84 t ha^−1^). This was associated with a 1-day shorter simulated duration from sowing to maturity.

## Data accessibility

Meteorological data from the Rothamsted Long-term Experiments are available at http://www.era.rothamsted.ac.uk.

Sirius, including calibrated cultivar parameters, is available from https://doi.org/10.5061/dryad.2v6wwpzmn.

Simulation model outputs for the data analysed in the paper are available at https://repository.rothamsted.ac.uk/item/9832z/simulated-wheat-yields-from-sirius-1892-2016, with code for the analysis as a R Markdown file available at https://repository.rothamsted.ac.uk/item/98330/reproducing-analysis-of-sirius-model-simulations-of-wheat-yields-1892-2016.
